# Electrospray deposition device used to precisely control the matrix crystal to improve the performance of MALDI MSI

**DOI:** 10.1038/srep37903

**Published:** 2016-11-25

**Authors:** Shilei Li, Yangyang Zhang, Jian’an Liu, Juanjuan Han, Ming Guan, Hui Yang, Yu Lin, Shaoxiang Xiong, Zhenwen Zhao

**Affiliations:** 1 Beijing National Laboratory for Molecular Sciences, Key Laboratory of Analytical Chemistry for Living Biosystems, Institute of Chemistry Chinese Academy of Sciences, Beijing Mass Spectrum Center, Beijing, 100190, P.R. China; 2 University of Chinese Academy of Sciences, Beijing, 100049, P.R.China

## Abstract

MALDI MSI has been recently applied as an innovative tool for detection of molecular distribution within a specific tissue. MALDI MSI requires deposition of an organic compound, known as matrix, on the tissue of interest to assist analyte desorption and ionization, in which the matrix crystal homogeneity and size greatly influence the imaging reproducibility and spatial resolution in MALDI MSI. In this work, a homemade electrospray deposition device was developed for deposition of matrix in MALDI MSI. The device could be used to achieve 1 μm homogeneous matrix crystals in MALDI MSI analysis. Moreover, it was found, for the first time, that the electrospray deposition device could be used to precisely control the matrix crystal size, and the imaging spatial resolution was increased greatly as the matrix crystals size becoming smaller. In addition, the easily-built electrospray deposition device was durable for acid, base or organic solvent, and even could be used for deposition of nanoparticles matrix, which made it unparalleled for MALDI MSI analysis. The feasibility of the electrospray deposition device was investigated by combination with MALDI FTICR MSI to analyze the distributions of lipids in mouse brain and liver cancer tissue section.

Matrix-assisted laser desorption ionization mass spectrometry imaging (MALDI MSI) has been widely used for *in situ* analysis of endogenous small molecular metabolites and exogenous drugs in tissue sections^1^,^2^,^3^,^4^. The most promising advantage of MALDI MSI is the reality of label-free detection, and to perform compound analysis avoiding extraction and/or separation steps and preserve the morphological integrity of analyzed tissues. MALDI MSI provides the location of biomolecules or drug and its metabolites within a specific tissue, which could be used for understanding the underlying mechanisms, the pharmacological or toxicological effects, etc.^5^,^6^,^7^,^8^,^9^. MALDI MSI has become a powerful imaging technology and is developing quickly.

MALDI MSI requires deposition of an organic compound, known as matrix, on the tissue of interest to assist analyte desorption and ionization^10^. In a typical MALDI MSI experiment, matrix solution is applied to a tissue slide surface that co-crystallized with the analyte forming analyte-matrix crystal across the surface of the tissue slide before MALDI MSI analysis^1^,^11^. The heterogeneous matrix crystals or an excessive amount of matrix could lead to the presence of so-called hot spots on the tissue sample. Meanwhile, the mass spectrometer instrument parameters, including raster step size and laser beam diameter, which could influence the spatial resolution of MALD MSI, are limited by the matrix crystal size^12^,^13^. Therefore, the matrix crystal homogeneity and size greatly influence the imaging reproducibility and spatial resolution in MALDI MSI^14^,^15^.

Currently, three matrix application methods, including airbrush, automatic sprayer and sublimation, are frequently used for depositing matrix^16^,^17^. The manually controlled airbrush is the most used matrix application method because of its simpleness, inexpensiveness and easy operation^18^; however, variations in the spray velocity and duration cause inconsistent application. The commercial automatic sprayer based on oscillating capillary nebulizer and inkjet printing can greatly improve matrix homogeneity, resulting in good matrix deposition repeatability^13^,^17^. Recently, it was reported that the automatic sprayer method could double the number of metabolites detected, and was more reproducible and less analyte diffusion than the airbrush method^17^. Sublimation matrix deposition yielded high spatial resolution and reproducibility but fewer analytes in the higher m/z range (500–1000 m/z). When the samples were placed in a humidity chamber after sublimation, there was enhanced detection of higher mass metabolites but increased analyte diffusion in the lower mass range^17^. Recently, an electric field-assisted matrix coating method was developed to deposit matrix on tissue with crystal sizes of <10 μm, which could enhance the detection of small molecule metabolites for MALDI MSI analysis by using N-(1-naphthyl) ethylenediamine dihydrochloride as matrix in negative ion mode^19^.

In this work, a homemade electrospray deposition device was developed for deposition of matrix in MALDI MSI. The parameters which greatly influenced the matrix crystal were optimized. Four scales of matrix 2, 5-dihydroxybenzoic acid (DHB) crystals with the size at 1, 10, 50 and 200 μm were prepared. It was found, for the first time, that the electrospray deposition device could be used to precisely control the matrix crystal size. More importantly, the DHB crystals with the size at 1 μm obviously improved the spatial resolution of MALD MSI. The device could also be used for other matrices deposition, including 9-aminoacridine (9-AA), α-cyano-4-hydroxycinnamic acid (CHCA), 2-Mercaptobenzothiazole (MBT) and TiO_2_ nanoparticles (TiO_2_ NPs). We further employed the device with MALDI Fourier transform ion cyclotron resonance mass spectrometry (MALDI FTICR MS) to investigate the distributions of lipids in mouse brain and liver cancer tissue.

## Results

### Electrospray deposition device for precisely controlling the matrix crystal

**Figure 1:**
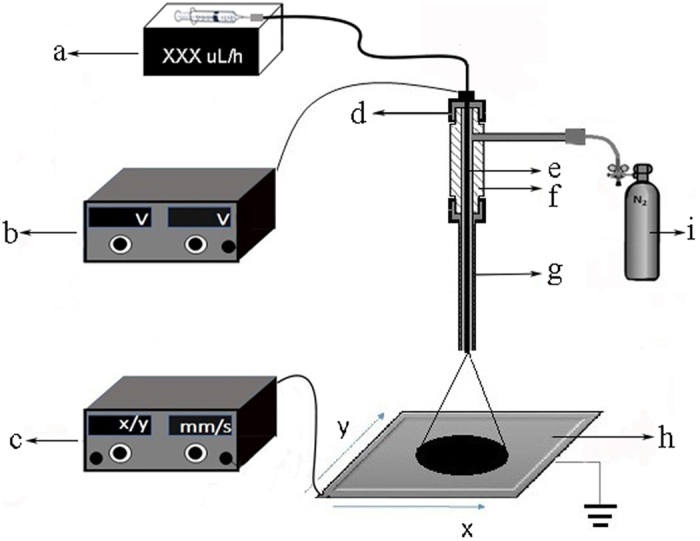
Schematic diagram of the homemade electrospray deposition device. a, Injection pump; b, High voltage device; c, motorized X−Y positioning stage controller; d, Teflon nut; e, Steel capillary (i.d. = 100 μm, o.d. = 150 μm); f, Teflon insulation shielding layer (i.d. = 400 μm); g, Teflon tube (i.d. = 400 μm; o.d. = 728 μm); h, mobile platform; i, Sheath gas.

**Figure 2:**
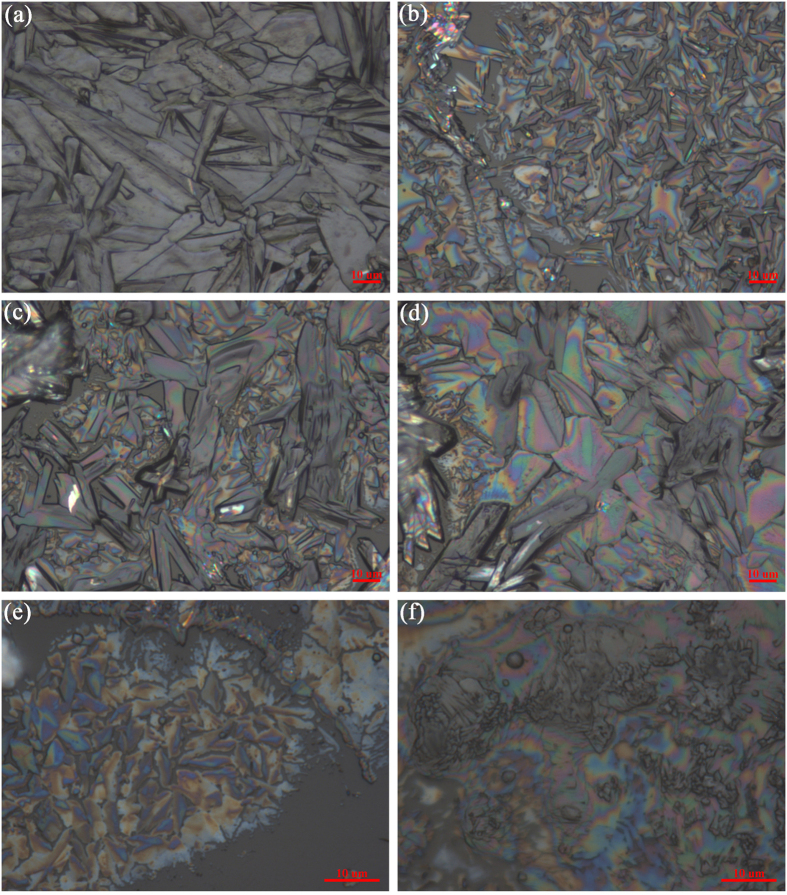
DHB crystal morphology under various working parameters listed in Table 1. Pictures were obtained by microscopy.

A schematic diagram of the homemade electrospray deposition device is shown in Fig. 1. The matrix solution was sprayed through a stainless steel capillary (i.d. = 100 μm). In this device, the electrospray voltage, the height of the spray tip above the ITO-slide, the solvent used, and the flow rate of matrix solution were very important parameters which greatly influenced the matrix crystal. In this study, these parameters were firstly investigated to precisely control the matrix crystal. Matrix DHB was firstly utilized to evaluate the performance of the homemade electrospray deposition device. A volume of 200 μL of DHB matrix solution with CH_3_CN:H_2_O as solvent was infused. The imaging results were shown in Fig. 2 and Table 1.

### The electrospray voltage

Fixing other parameter, it was found that the DHB crystal became smaller under high voltage condition (Fig. 2b and d).

### The height of the spray tip above the ITO-slide

Fixing other parameter, we found that the scales of the DHB-analyte crystals were becoming smaller from 50 to 10 μm as the distance increasing from 4.5 to 8.5 cm (Fig. 2a and b). On the other side, the DHB-analyte crystals were becoming slightly bigger as the distance increasing from 8.5 to 10.5 cm (Fig. 2b and c). We inferred that there was correlation between distance and nebulization efficiency. From 4.5 to 8.5 cm, the nebulization efficiency was increased, however, the electric filed was decreased as the distance from 8.5 to 10.5 cm, and therefore, the DHB-analyte crystals were becoming slightly bigger. On the other side, we have found the spraying diameter on the ITO-slide slightly increased as the distance increasing.

### The solvent used

High percent organic solvent easily generate smaller matrix crystal; however, it also led to blocking in matrix application. On the basis of our experiences for DHB deposition, two organic solvent percentages were tested: CH3CN:H2O = 8:2 and CH3CN, which was shown that the higher portion of CH3CN led to the smaller crystals, but higher portion of CH3CN easily led to heterogeneity due to the droplets drying in transit (Fig. 2b and f).

### The flow rate of matrix solution

It was shown that that the DHB crystal became smaller indeed with the matrix solution flowing rate decreasing from 250 to 125 μL/h. However, the co-crystal became heterogeneous (Fig. 2b and e). The reason is that some crystals were formed before reaching the tissue surface. In addition, decreasing the flow rate will spent more time to deposit the matrix.

**Figure 3:**
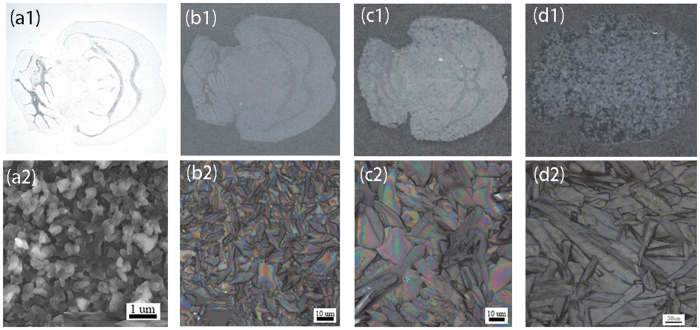
Four scales of matrix DHB crystals prepared by the homemade electrospray deposition device under the four conditions listed inTable 2. (**a1**) 1 μm; (**b1**) 10 μm; (**c1**) 50 μm; (**d1**) 200 μm. Pictures of (**a1,b1,c1** and **d1**) were obtained by a scanner (HP LaserJet M1216nfh MFP). Pictures of (**a2,b2,c2** and **d2**) were high resolution images of (**a1,b1,c1** and **d1**), respectively. The (**a2**) was obtained by scanning electron microscopy (SEM JEOL 6701) and (**b2,c2,d2**) were obtained by microscopy.

In summary, the size of matrix crystals was a result of comprehensive factors. By adjusting these parameters, shown in Table 2, we successfully prepared four scales of DHB crystals with the size at 1, 10, 50 and 200 μm, shown in Fig. 3. It was observed under microscopy that the crystal morphology was very homogenous. The final deposition density was 0.10–0.15 mg/cm^2^ (Table 2) due to the different spraying square. In addition, the reproducibility and repeatability experiments were performed. The results were shown in Figs S2 and S3. From the results, we can see that similar DHB crystals were obtained from different time with the same deposition parameters (Fig. S2). In addition, the MSI results for the mouse brain were also repeatable (Fig. S3).

**Figure 4:**
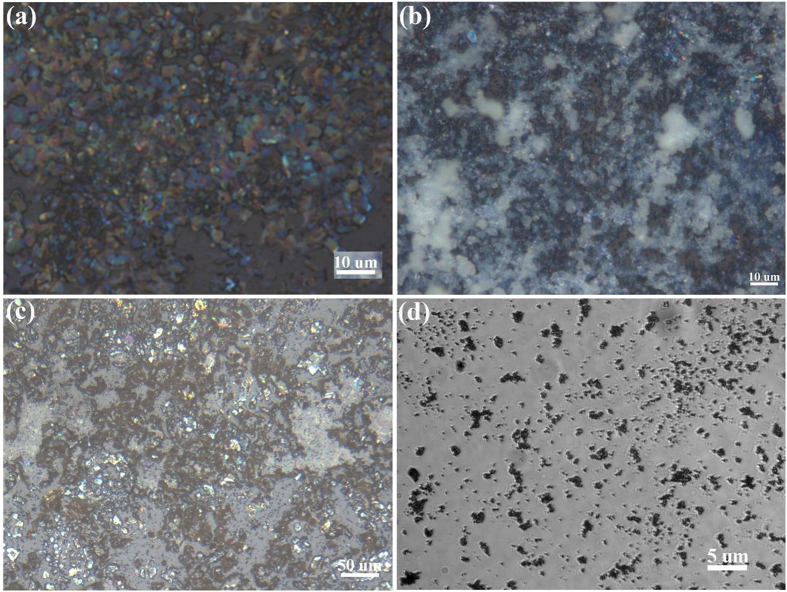
The crystal morphology of matrix 9-AA (**a**), CHCA (**b**), MBT (**c**), and TiO_2_ NPs (**d**) prepared by the homemade electrospray deposition device under the four conditions listed in Table 3. Pictures were obtained by microscopy.

The parameters for other matrices deposition, including 9-AA, CHCA, MBT and TiO_2_ NPs, were also investigated. Under the conditions shown in Table 3, less than 10 μm 9-AA, CHCA and MBT crystals were obtained, and each TiO_2_ NPs aggregate was even less than 1 μm, shown in Fig. 4. Taken together, the homemade electrospray deposition device could be used to achieve 1 μm homogeneous matrix crystals to support the 10 μm spatial resolution in MALDI MSI analysis. In addition, the homemade electrospray deposition device was durable for acid, base or organic solvent, and would not be blocked easily, even could be used for deposition of nanoparticles; moreover, the device was easy to be built and cleaned, which made it unparalleled for MALDI MSI analysis.

### The effect of matrix crystal on the MALDI MSI analysis

**Figure 5:**
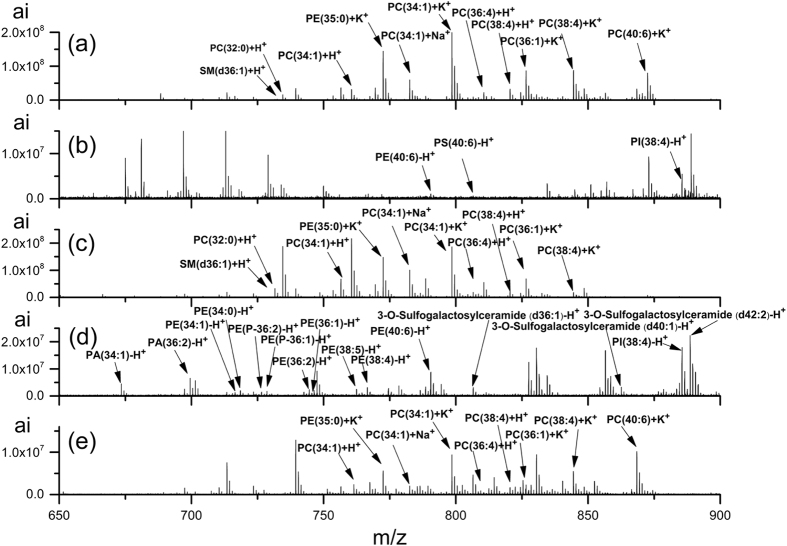
Mass spectra of mouse brain tissue section obtained by MALDI FTICR MSI using DHB (**a** and **b**), MBT (**c** and **d**), and TiO2 NPs (**e**) as a matrix, respectively. In (**a**,**c** and **e**), the positive ion mode of mass spectrometer was used, while in (**b** and **d**), the negative ion mode of mass spectrometer was used. ai, absolute intensity.

**Figure 6:**
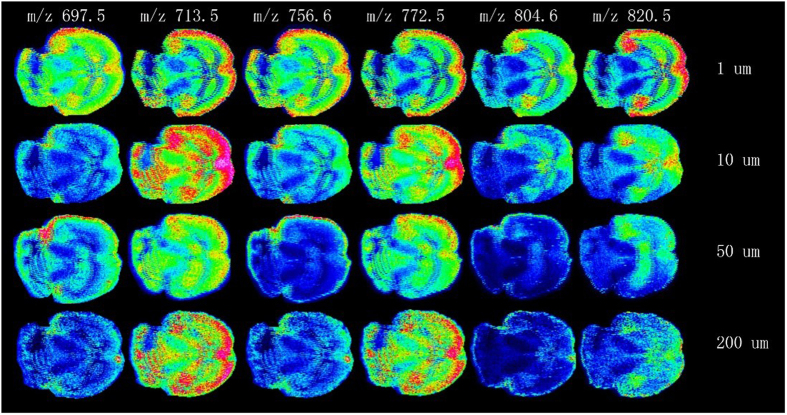
The representative ions images in brain tissue section obtained by MALDI FTICR MSI by deposition four sizes of matrix DHB crystals: 1 μm, 10 μm, 50 μm, 200 μm. The raster step size and laser beam diameter were 100 μm and 25 μm, respectively.

The effect of four scales of DHB crystals with the size at 1, 10, 50 and 200 μm on the desorption and ionization of analyte in MALDI MSI analysis was firstly investigated. Shown in Fig. 5a and b were mass spectra of mouse brain tissue section obtained by MALDI FTICR MSI using DHB as a matrix in the positive ion mode and negative ion mode, respectively, regardless of DHB crystals size. All the four scales of DHB crystals exhibited strong ability for desorption and ionization of phosphatidylcholines (PCs) in the positive ion mode, and few phosphatidylthanolamines (PEs), phosphatidylinositols (PIs) and phosphatidylserine (PSs) were detected in the negative ion mode. The only difference is that, when the DHB crystals size became smaller, the laser energy needed for desorption and ionization of analyte was lower, which could protect the laser equipment of mass spectrometer. Further, the effect of four scales of DHB crystals with the size at 1, 10, 50 and 200 μm on the spatial resolution of MALD MSI analysis was investigated. The representative ions images in brain tissue section obtained by MALDI FTICR MSI by deposition four sizes of matrix DHB crystals (1 μm, 10 μm, 50 μm and 200 μm) were shown in Fig. 6. It was observed that the specific tissues in mouse brain, such as cortex, hippocampus and cerebellum could be distinguished clearly by the results of MALDI FTICR MSI analysis when DHB crystals with the size at 1 μm were used. In general, the imaging resolution was increased obviously as the DHB crystals size becoming smaller. Currently, The Image PrepTM matrix application instrument (Bruker Inc., Germany) is wide used for matrix deposition; therefore, we made a comparison with it. The imaging results, shown in Fig. S3, demonstrated the advantage of our homemade electrospray deposition device, in terms of good sensitivity and spatial resolution. Moreover, our homemade electrospray deposition device is time-saving and matrix-saving.

In addition, the effect of other matrices on the desorption and ionization of analyte in MALDI MS analysis of mouse brain tissue section was investigated as well. By using the homemade electrospray deposition device, less than 10 μm size of 9-AA, CHCA, MBT and TiO_2_ NPs crystals were prepared and already shown in Fig. 4. In the positive ion mode, 9-AA (data not shown), CHCA (data not shown), MBT (shown in Fig. 5c) and TiO_2_ NPs (shown in Fig. 5e), all of them, exhibited strong ability for desorption and ionization of PCs, and their mass spectra were very similar. There is a quaternary amine in the PC structure, which may lead to PC being detected conveniently in positive ion mode. However, in the negative ion mode, only 9-AA (data not shown) and MBT (shown in Fig. 5d) showed good ability for desorption and ionization of analyte, and lots of phosphatidylacids (PAs), PEs, PIs and PSs were detected. Using CHCA and TiO_2_ NPs as matrix, no signal was detected in the negative ion mode. Taken together, in MALDI MSI analysis, the matrix is still a very critical factor, and the matrix 9-AA, CHCA, MBT and TiO_2_ NPs crystals prepared by the homemade electrospray deposition device should be specifically chosen for MALDI MSI analysis of different targeted analytes.

### The application of the homemade electrospray deposition device in MALDI MSI analysis

**Figure 7:**
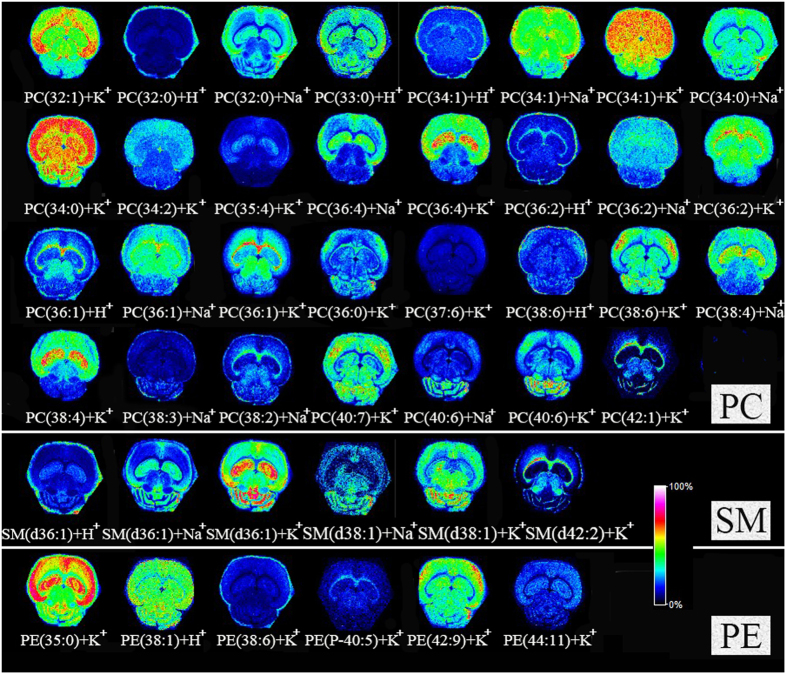
The lipids distribution in mouse brain tissue section obtained by MALDI FTICR MSI by deposition 1 μm size of matrix DHB crystals. The raster step size and laser beam diameter were 100 μm and 25 μm, respectively.

To examine the feasibility of the electrospray deposition device, we employed mouse brain tissue sections as one of our model samples for MALDI FTICR MSI analysis. The matrix DHB with crystal size at 1 μm was deposited on the tissue sections. Many lipids, including PCs, sphingomylins (SMs) and PEs were detected. The high-resolution MS spectra were used to distinguish different compounds, and the identification of compounds was achieved by precisely matching mass with free online databases HMDB (http://www.hmdb.ca) (accessed July 2015)^20^,^21^,^22^. The mass error was set at 5 ppm. In addition, MS/MS spectra obtained by collision induced dissociation (CID) were further used for confirmation of the structure of the compounds. The m/z detected, the theoretical m/z, the mass error, and the MS/MS spectra of representative ions were shown in Table S1 and Fig. S4. The lipids distribution in mouse brain tissue section obtained by MALDI FTICR MSI was shown in Fig. 7. From the images, it could be observed that the distributions of these lipids detected in the mouse brain tissue section were very sharp, which could be used to reveal key insights into their roles and functions within the organism.

**Figure 8:**
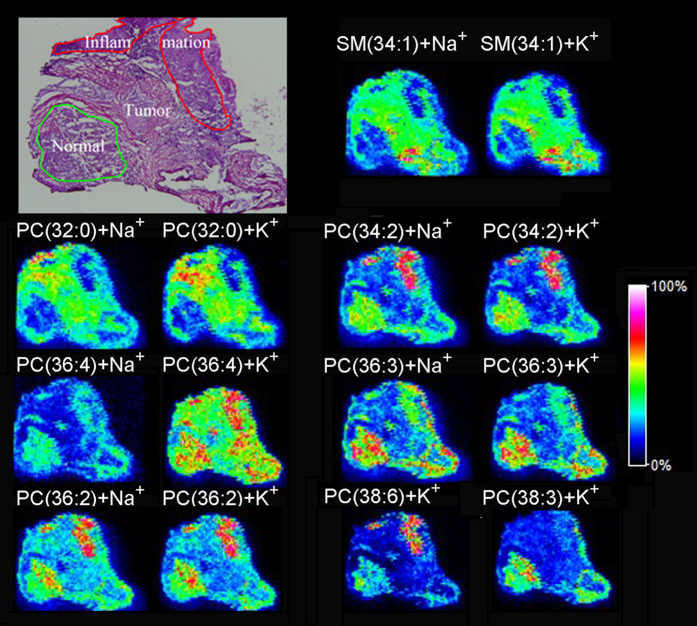
The histological morphology imaging by H&E staining and lipids distribution in liver cancer tissue section obtained by MALDI FTICR MSI. The raster step size and laser beam diameter were 100 μm and 25 μm, respectively.

The human liver cancer tissue, as another example, was analyzed as well by MALDI FTICR MSI. The matrix DHB with crystal size at 1 μm was also deposited on the tissue sections. The histological morphology imaging by H&E staining and lipids distribution in liver cancer tissue section obtained by MALDI FTICR MSI was shown in Fig. 8. Interestingly, SM (34:1) and PC (32:0), in which there were 1 or 0 double bond in these lipids structure, were highly expressed, while PC (34:2), PC (36:4), PC (36:3), PC (36:2), PC (38:6) and PC (38:3), in which there were 2 or more than 2 double bonds in these lipids structure, were down regulated in tumor area. The unique distribution of these lipids were well corresponding to the histological morphology imaging. Although the reason for these lipid molecules that are either elevated or attenuated in liver tissue is not clear yet, the analyses have significantly expanded our knowledge related to human physiology and pathology, and could engender new insights into cancer pathobiology.

## Discussion

In this work, a homemade electrospray deposition device was developed for deposition of matrix in MALDI MSI. Four scales of DHB crystals with the size at 1, 10, 50 and 200 μm were successfully prepared by the device. The device was also feasible for deposition of other organic compound matrix, such as 9-AA, CHCA and MBT, and even could be used for deposition of nanoparticles matrix, like TiO_2_ NPs. The matrix crystals were homogenous, and more importantly, the matrix crystal was controllable. Less than 10 μm DHB, 9-AA, CHCA and MBT crystals were obtained, and each TiO_2_ NPs aggregate was even less than 1 μm. It was found, for the first time, that the imaging spatial resolution was increased greatly as the matrix crystals size becoming smaller. The lipids distribution in mouse brain and human liver cancer tissue section were analyzed by combining the device with MALDI FTICR MS. By deposition of matrix DHB with crystal size at 1 μm, the distributions of lipids detected in the mouse brain and liver cancer tissue section were very sharp, which could be used to reveal key insights into their roles and functions within the organism. The homemade electrospray deposition device was durable for acid, base or organic solvent, and easy to be built and cleaned, which made it unparalleled for MALDI MSI analysis.

## Methods

### Materials and reagents

The matrix 2,5-dihydroxybenzoic acid (DHB) and 2-Mercaptobenzothiazole (MBT) were purchased from Lancaster (Morecambe, UK). The matrix α-cyano-4-hydroxycinnamic acid (CHCA) and 9-aminoacridine (9-AA) were purchased from Acros (New Jersey, US). TiO_2_ nanoparticles (TiO_2_ NPs) were purchased from Sigma-Aldrich (St. Louis, MO). HPLC-grade methanol (MeOH) and acetonitrile (CH_3_CN) were purchased from Fluka Feinchemikalien GmbH (part of Sigma−Aldrich Chemie GmbH, Taufkirchen, Germany). All of the above materials were used as received without further purification.

### Electrospray deposition device for deposition of matrix

A schematic diagram of the homemade electrospray deposition device was shown in Fig. 1. The physical photo of the homemade electrospray deposition device was shown in Fig. S1 in the Supporting Information. The matrix DHB was dissolved in CH_3_CN:H_2_O, 9-AA, CHCA, and MBT were dissolved in MeOH, and TiO_2_ NPs was dissolved in H_2_O. For deposition of matrix, the matrix solution was sprayed through a stainless steel capillary (i.d. = 100 μm). The slide was mounted on a motorized X−Y positioning stage with the movement speed was at 1 mm/s. The flow rate of sheath gas was 50 L/h. The working parameters were optimized, and showed in Tables 2 and 3. The duration for matrix coating of mouse brain and liver cancer tissue section is 30 or 60 min.

### Tissue samples

The C57BL/6 mice (5 months old, Female) were provided by Dr. Weidong Yong in the institute of Laboratory Animal Science, Chinese Academy of Medical Sciences. The mice were sacrificed by suffocation of CO_2_, and their brains were immediately surgically removed and quickly frozen in liquid nitrogen. These tissues were stored at −80 °C until use. The animal experiments were performed according to “the Guide for the Care and Use of Laboratory Animals” and were approved by the Animal Care and Use Committee of the Chinese Academy of Sciences. The liver cancer tissues were obtained from patients undergoing cancer resection at Peking University People’s Hospital. The ethical approval of the present study was obtained from the ethical committee of Peking University People’s Hospital (Beijing, China). The methods were carried out in accordance with the approved guidelines. Tissue samples were collected in tubes, quickly frozen using liquid nitrogen and stored at −80 °C until use. The project was approved by the Institutional Review Board, and written informed consent forms were signed by participants.

### Tissue sectioning

Frozen mouse brain or human liver cancer tissue was fixed atop a drop of saline on the cutting stage. All tissues were sectioned at 12 μm thickness using a Leica CM1950 cryostat (Leica Microsystems GmbH, Wetzlar, Germany) at −18 °C and thaw mounted onto indium tin oxide (ITO) coated glass slides (Type Ι 1.1 mm/100ea, HST Inc., Newark, NJ, USA). The glass slides were then placed into a vacuum desiccator and dried for approximately 1 h before matrix application. Finally, matrix was sprayed on the tissue section by homemade electrospray deposition device. The tissue section was used for MALDI MSI analysis. Hematoxylin and eosin (H&E) staining was also performed to obtain histological morphology imaging.

### MALDI-FTICR mass spectrometry

MALDI-FTICR mass spectrometric analysis was performed with a Bruker solariX mass spectrometer equipped with a 9.4 T superconducting magnet and SmartBeam™ laser optics. Mass calibrations were performed externally using DHB and a peptide mixture (angiotensin II, substance P, bombesin, and ACTH clip 1–17) (Sigma, St. Louis, MO, USA) as mass standards. External ion accumulation was used in both positive and negative ion mode over a mass range of 200–1800 m/z with a resolution of 200,000 at m/z 200. SolariX Control software was used for data acquisition. For MALDI MSI analysis, mass spectra were acquired across the entire sample section with a SmartBeam II laser operating at 1000 Hz, a laser focus of 25 μm, 1 scan acquired from each matrix spot, and a raster step size of 100 μm. The device parameters for MALDI MSI were chosen as follows: plate offset voltage, 100 V; deflector plate voltage, 180 V. Data were processed using DateAnalysis 4.0 (Bruker Daltonics) and FlexImaging 3.0 software (Bruker Daltonics).

### Structural identification

High-resolution MS spectra were used to distinguish different compounds, and the identification of compounds was achieved by precisely matching mass with free online databases HMDB (http://www.hmdb.ca) (accessed July 2015)^20^,^21^,^22^. The mass error was set at 5 ppm. In addition, MS/MS spectra obtained by collision induced dissociation (CID) were further used for confirmation of the structure of the compounds.

## Additional Information

**How to cite this article**: Li, S. *et al*. Electrospray deposition device used to precisely control the matrix crystal to improve the performance of MALDI MSI. *Sci. Rep.* **6**, 37903; doi: 10.1038/srep37903 (2016).

**Publisher’s note:** Springer Nature remains neutral with regard to jurisdictional claims in published maps and institutional affiliations.

## Supplementary Material

Supplementary Information

## Figures and Tables

**Table 1:** Working parameters for obtaining the different sizes of DHB crystals in Fig. 2.

	Height (cm)	High voltage (v)	Injection rate (μL/h)	Solvent (CH_3_CN:H_2_O)	Concentration (mg/mL)	Deposition time (min)	Sheath gas (L/h)	Matrix deposition density (mg/cm^2^)
a	4.5	5000	250	8:2	25	12	50	0.05
b	8.5	5000	250	8:2	25	12	50	0.04
c	10.5	5000	250	8:2	25	12	50	0.03
d	8.5	*NA*	250	8:2	25	12	50	0.06
e	8.5	5000	125	8:2	25	24	50	0.04
f	8.5	5000	250	10:0	25	12	50	0.04

Height: the distance of the spray tip above the ITO-slide.

*NA: not available.*

**Table 2:** Four optimizing working parameters for obtaining four sizes of DHB crystals.

Size of DHB crystal	Height (cm)	High voltage (v)	Injection rate (μL/h)	Solvent (CH_3_CN:H_2_O)	Concentration (mg/mL)	Deposition time (min)	Sheath gas (L/h)	Matrix deposition density(mg/cm^2^)
1 μm	1.5	5000	250	10:0	40	20	0	0.14
10 μm	8.5	5000	250	8:2	25	30	50	0.10
50 μm	8.5	*NA*	250	8:2	25	30	50	0.15
200 μm	8.5	*NA*	500	8:2	25	15	50	0.15

Height: the distance of the spray tip above the ITO-slide.

*NA: not available.*

**Table 3:** Optimized parameters of the homemade electrospray deposition device for deposition of matrix 9-AA, CHCA, MBT and TiO_2_ NPs, respectively.

Matrix	Height (cm)	High voltage (V)	Injection rate (uL/h)	Solvent	Concentration (mg/mL)	Deposition time (min)	Sheath gas (L/h)	Matrix deposition density (mg/cm^2^)
9-AA	8.5	5000	250	MeOH	20	30	50	0.08
CHCA	8.5	5000	250	MeOH	10	60	50	0.08
MBT	8.5	5000	250	MeOH	20	30	50	0.08
TiO_2_ NPs	8.5	5000	250	H_2_O	2	30	50	0.008

Height: the distance of the spray tip above the ITO-slide.
