# Supplementary material for: Electrospray deposition device used to precisely control the matrix crystal to improve the performance of MALDI MSI
Source: Sci Rep. 2016 Nov 25;6:37903. doi: 10.1038/srep37903 (PMC5122855; doi:10.1038/srep37903)
Supplement: Supplementary Information [file srep37903-s1.pdf]

Electrospray deposition device used to precisely control the matrix crystal to improve the performance of MALDI MSI

Shilei Li[1,2], Yangyang Zhang[1], Jian'an Liu[1], Juanjuan Han[1], Ming Guan[1,2], Hui Yang[1,2], Yu Lin[1], Shaoxiang Xiong[1] & Zhenwen Zhao[1,2]

[1] Beijing National Laboratory for Molecular Sciences, Key Laboratory of Analytical Chemistry for Living Biosystems, Institute of Chemistry Chinese Academy of Sciences, Beijing Mass Spectrum Center, Beijing, 100190, P.R. China. [2] University of Chinese Academy of Sciences, Beijing, 100049, P.R.China. Correspondence and requests for materials should be addressed to Z.Z. (email: zhenwenzhao@iccas.ac.cn)

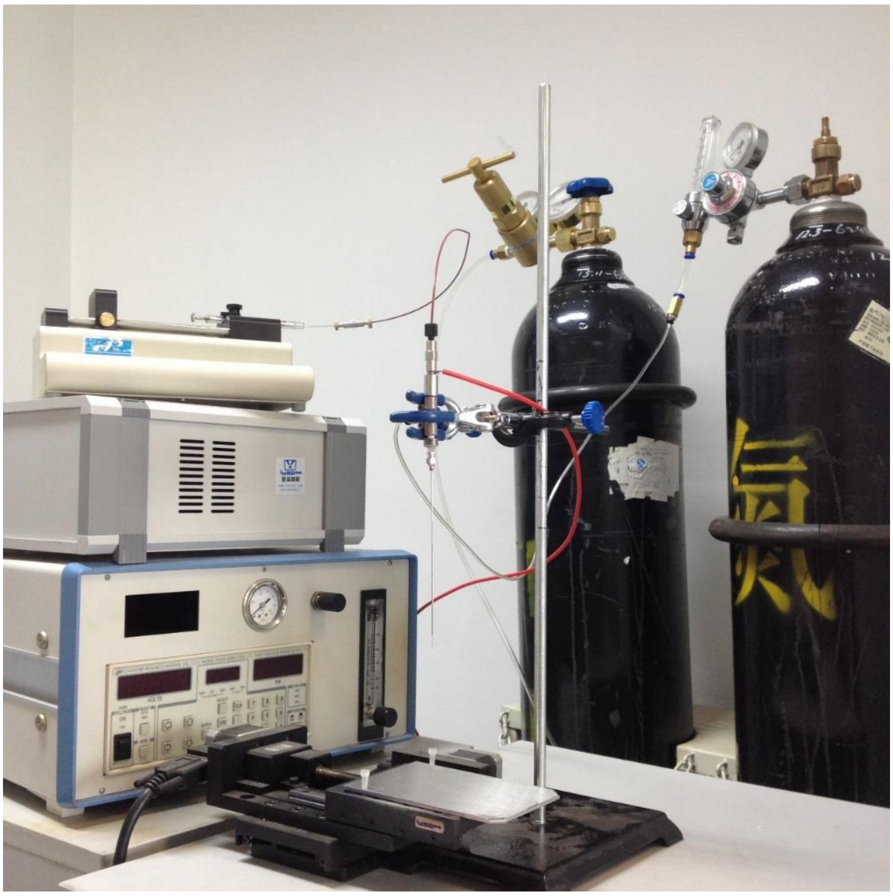

Fig. S1 The physical photo of the homemade electrospray deposition device.

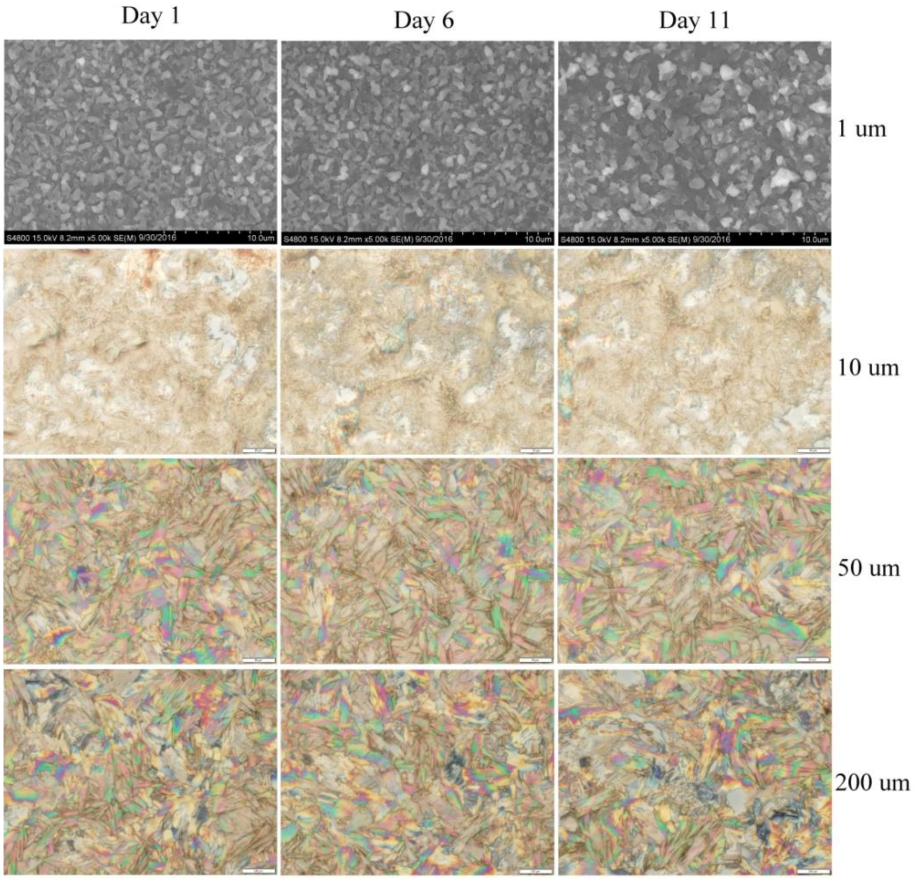

Fig. S2 DHB crystal morphology which was produced in day 1, 6 and 11 under the working parameters listed in Table 1 in our manuscript, respectively.

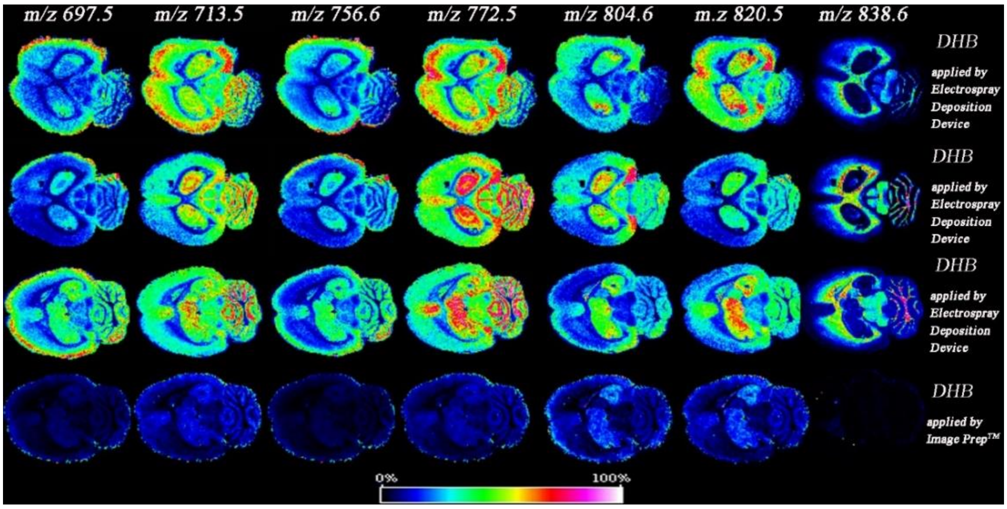

Fig. S3 The representative ions images in brain tissue section obtained by MALDI FTICR MSI by using our homemade electrospray deposition device three times and Image Prep^{TM} one time for DHB deposition, respectively. The raster step size and laser beam diameter were 200 µm and 25 µm, respectively.

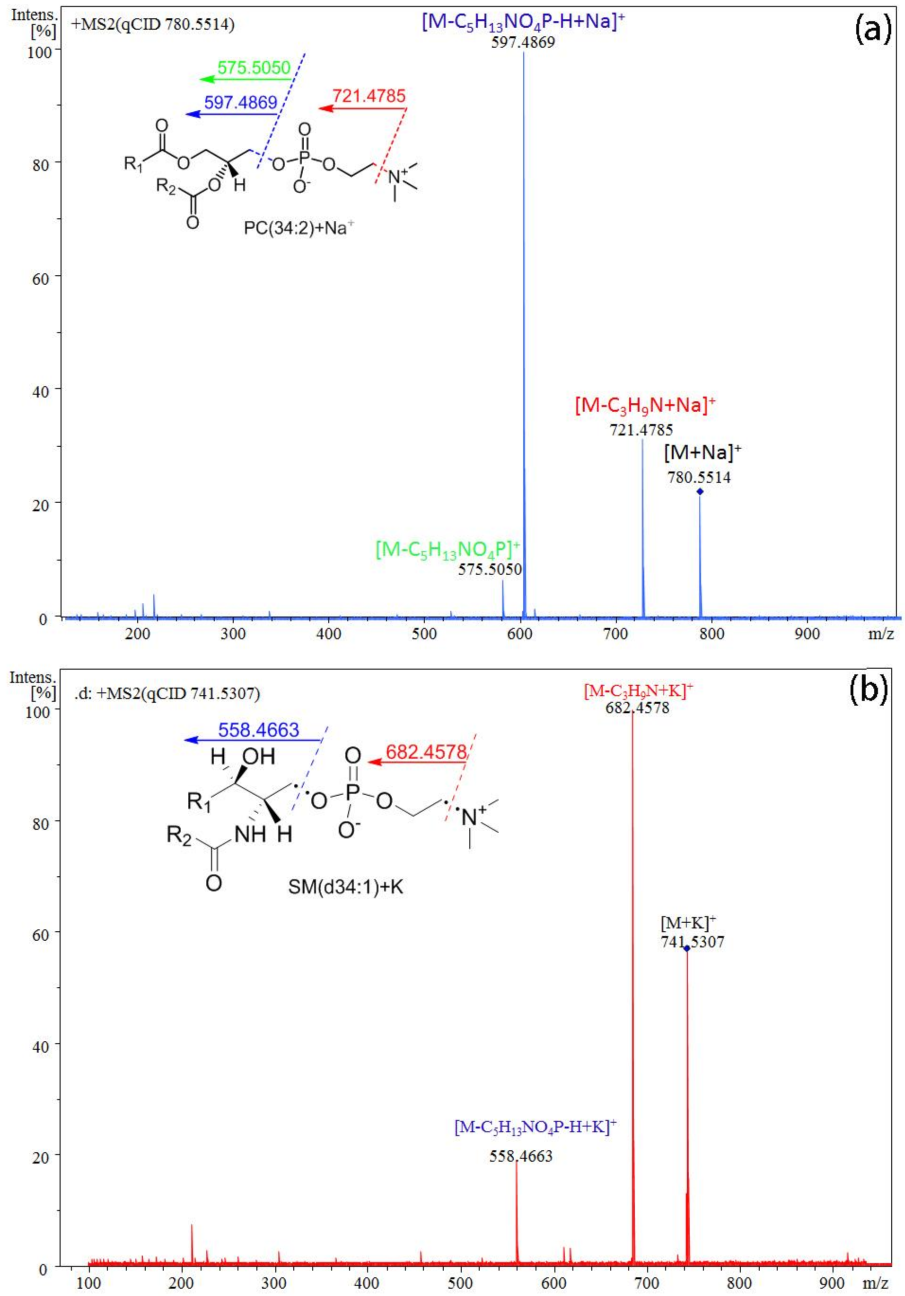

Fig. S4 The MS/MS spectra of representative PC (a) and SM (b) in positive ion mode by MALDI FTICR MS.

Table S1 Lipids detected in mouse brain tissue section by MALDI FTICR MS in positive ion mode.

Species	No.	m/z detected	Database search result	Name	Ion type	m/z exact	Mass accuracy /ppm
PC	1	770.5112	HMDB08064	PC(32:1)	$M+K^+$	770.5097	1.9948
	2	734.5685	HMDB07871	PC(32:0)	$M+H^+$	734.5694	1.2674
		756.5514	HMDB07871	PC(32:0)	$M+Na^+$	756.5514	0.0357
	3	748.5857	HMDB07937	PC(33:0)	$M+H^+$	748.5851	0.8269
	4	760.5849	HMDB08263	PC(34:1)	$M+H^+$	760.5851	0.2380
		782.5670	HMDB08263	PC(34:1)	$M+Na^+$	782.5670	0.0294
		798.5408	HMDB08263	PC(34:1)	$M+K^+$	798.5410	0.2041
	5	784.5817	HMDB07970	PC(34:0)	$M+Na^+$	784.5827	1.2401
		800.5536	HMDB07970	PC(34:0)	$M+K^+$	800.5566	3.7636
	6	796.5239	HMDB08296	PC(34:2)	$M+K^+$	796.5253	1.7740
	7	806.5134	HMDB07949	PC(35:4)	$M+K^+$	806.5097	4.6335
	8	804.5513	HMDB08623	PC(36:4)	$M+Na^+$	804.5514	0.0907
		820.5247	HMDB08623	PC(36:4)	$M+K^+$	820.5253	0.7471
	9	786.6034	HMDB00593	PC(36:2)	$M+H^+$	786.6007	3.3931
		808.5823	HMDB00593	PC(36:2)	$M+Na^+$	808.5827	0.4613
		824.5565	HMDB00593	PC(36:2)	$M+K^+$	824.5566	0.1370
	10	788.6166	HMDB08558	PC(36:1)	$M+H^+$	788.6164	0.2777
		810.5988	HMDB08558	PC(36:1)	$M+Na^+$	810.5983	0.5885
		826.5725	HMDB08558	PC(36:1)	$M+K^+$	826.5723	0.2867
	11	828.5877	HMDB07886	PC(36:0)	$M+K^+$	828.5879	0.2571
	12	830.5099	HMDB07958	PC(37:6)	$M+K^+$	830.5097	0.2854
	13	806.5690	HMDB07991	PC(38:6)	$M+H^+$	806.5694	0.5344
		844.5254	HMDB07991	PC(38:6)	$M+K^+$	844.5253	0.1030
	14	832.5824	HMDB08145	PC(38:4)	$M+Na^+$	832.5827	0.3279
		848.5574	HMDB08145	PC(38:4)	$M+K^+$	848.5566	0.9275
	15	834.5987	HMDB08594	PC(38:3)	$M+Na^+$	834.5983	0.4517
	16	836.6113	HMDB07927	PC(38:2)	$M+Na^+$	836.6140	3.1950
	17	870.5404	HMDB08090	PC(40:7)	$M+K^+$	870.5410	0.6467
	18	856.5814	HMDB08727	PC(40:6)	$M+Na^+$	856.5827	1.4861
		872.5562	HMDB08727	PC(40:6)	$M+K^+$	872.5566	0.4733
	19	910.6693	HMDB08315	PC(42:1)	$M+K^+$	910.6662	3.4436
SM	1	731.6058	HMDB12088	SM(d36:1)	$M+H^+$	731.6062	0.4798
		753.5874	HMDB12088	SM(d36:1)	$M+Na^+$	753.5881	0.9196
		769.5616	HMDB12088	SM(d36:1)	$M+K^+$	769.5620	0.5627
	2	781.6174	HMDB12102	SM(d38:1)	$M+Na^+$	781.6194	2.5498
		797.5905	HMDB12102	SM(d38:1)	$M+K^+$	797.5933	3.5519
	3	851.6382	HMDB12107	SM(d42:2)	$M+K^+$	851.6403	2.4459
PE	1	772.5254	HMDB09219	PE(35:0)	$M+K^+$	772.5253	0.1126
	2	774.6015	HMDB09485	PE(38:1)	$M+H^+$	774.6007	0.9928
	3	802.4775	HMDB09294	PE(38:6)	$M+K^+$	802.4784	1.0754

	4	816.5283	HMDB09611	PE(P-40:5)	$M+K^+$	816.5304	2.5694
	5	852.4973	HMDB09471	PE(42:9)	$M+K^+$	852.4940	3.8557
	6	876.4942	HMDB09639	PE(44:11)	$M+K^+$	876.4940	0.2134
